# Speaker Counting Based on a Novel Hive Shaped Nested Microphone Array by WPT and 2D Adaptive SRP Algorithms in Near-Field Scenarios

**DOI:** 10.3390/s23094499

**Published:** 2023-05-05

**Authors:** Ali Dehghan Firoozabadi, Pablo Adasme, David Zabala-Blanco, Pablo Palacios Játiva, Cesar Azurdia-Meza

**Affiliations:** 1 Department of Electricity, Universidad Tecnológica Metropolitana, Av. José Pedro Alessandri 1242, Santiago 7800002, Chile; 2 Electrical Engineering Department, Universidad de Santiago de Chile, Av. Victor Jara 3519, Santiago 9170124, Chile; 3 Department of Computing and Industries, Universidad Católica del Maule, Talca 3466706, Chile; 4 Department of Electrical Engineering, Universidad de Chile, Santiago 8370451, Chile; 5 Escuela de Informática y Telecomunicaciones, Universidad Diego Portales, Santiago 8370190, Chile

**Keywords:** speech processing, speaker counting, source localization, adaptive processing, microphone arrays, classification, spectral estimation

## Abstract

Speech processing algorithms, especially sound source localization (SSL), speech enhancement, and speaker tracking are considered to be the main fields in this application. Most speech processing algorithms require knowing the number of speakers for real implementation. In this article, a novel method for estimating the number of speakers is proposed based on the hive shaped nested microphone array (HNMA) by wavelet packet transform (WPT) and 2D sub-band adaptive steered response power (SB-2DASRP) with phase transform (PHAT) and maximum likelihood (ML) filters, and, finally, the agglomerative classification and elbow criteria for obtaining the number of speakers in near-field scenarios. The proposed HNMA is presented for aliasing and imaging elimination and preparing the proper signals for the speaker counting method. In the following, the Blackman–Tukey spectral estimation method is selected for detecting the proper frequency components of the recorded signal. The WPT is considered for smart sub-band processing by focusing on the frequency bins of the speech signal. In addition, the SRP method is implemented in 2D format and adaptively by ML and PHAT filters on the sub-band signals. The SB-2DASRP peak positions are extracted on various time frames based on the standard deviation (SD) criteria, and the final number of speakers is estimated by unsupervised agglomerative clustering and elbow criteria. The proposed HNMA-SB-2DASRP method is compared with the frequency-domain magnitude squared coherence (FD-MSC), i-vector probabilistic linear discriminant analysis (i-vector PLDA), ambisonics features of the correlational recurrent neural network (AF-CRNN), and speaker counting by density-based classification and clustering decision (SC-DCCD) algorithms on noisy and reverberant environments, which represents the superiority of the proposed method for real implementation.

## 1. Introduction

Speech signal processing is considered to be a principal area in the intelligentization of robot–human communication systems [1]. The interface between humans and robots is used in applications such as human rescue, war and defense environments, earthquakes, unexpected events, factories, smart control systems, etc. Therefore, a wide range of signal processing techniques are included in this area such as simultaneous sound source localization (SSL) [2], speaker tracking [3], speech signal enhancement [4], speaker identification [5], voice activity detection [6], speaker counting [7], etc. Speaker tracking and source localization are two common speech processing algorithms, which are widely used in intelligent room implementations. Speech overlaps and fast changes between active speakers are important challenges in localization and tracking systems. Most of the localization algorithms require knowledge of the correct number of speakers for calculating accurate speaker locations. Speaker counting methods are implemented as a preprocessing step for many other speech processing algorithms. Therefore, the accuracy of speaker counting algorithms directly affects the precision of the speech processing methods. Speaker counting methods are highly affected by environmental conditions such as noise, reverberation, environmental temperature, etc. [8]. Methods that properly estimate the number of speakers (NoSs) must calculate the correct number of speakers in the worst acoustical scenarios. Aliasing and imaging are other factors that decrease the precision of NoS estimating methods because of inter-sensor distances. Microphone arrays provide the proper information about different indoor environments for signal processing due to the high number of sensors, but aliasing and imaging, because of the inter-sensor distance, affect the frequency components of recorded speech signals [9]. Then, proposing the microphone array with a proper and specific structure of the sensors increases the accuracy of the NoS estimating methods, which is one of the areas of interest in this research work.

In the last decade, various research works have been proposed for smart meeting room applications. Smart rooms include different types of microphone arrays with high quality microphones to provide the proper information for audio processing algorithms. In the cocktail party scenario, microphones record the overlapped speech signals of simultaneous speakers. Knowing the correct NoS is a common assumption in a speech processing system with various applications. This assumption is an important factor in the performance of speech signal processing. In common speaker separation algorithms [10], traditional systems do not directly provide information about the number of speakers. Therefore, estimating the NoS is required for eliminating the gap between theoretical and practical systems in real environments. In recent years, a few methods for estimating the NoS were proposed based on microphone arrays. In theory, estimating the NoS is directly related to the speaker identification application, which is known as speaker diarization [11,12]. If a system shows who is speaking in a specific time, it naturally provides the information about the number of simultaneous speakers in the overlapped speech signal area, which is known as counting by detection. A diarization system with high accuracy detects the number of speakers efficiently. In addition, the available diarization systems are implemented with a clear segmentation process, where the first step in this system is detecting the segments with one active speaker. The segments’ borders are detected by speaker change detectors [13]. The detected segments are selected for identifying the speakers’ locations of the recorded speech signals. The available segmentation methods fail in the cocktail party scenario when there are simultaneous active speakers. In fact, the overlapped segments in the speech signal are the main error source in diarization systems [14]. Some algorithms were proposed based on detecting and rejection of the overlapped speech segments for improving the performance of detection-based methods [15]. Detecting these segments is introduced as a research field in speech processing due to a wide range of proposed systems [16,17]. Finding the overlapped speech segments can be considered as a binary version of the NoS estimating challenge, where the number of overlapped speakers is one for non-overlapped signals and more than one for overlapped speech. Therefore, the NoS estimating methods can detect the overlapped speech segments but not vice versa. In addition, an overlapped speech detection system cannot be easily considered as a speaker separation algorithm. In fact, before using deep learning algorithms in speaker separation, the models require a high context, and in such cases as non-negative matrix factorization, the number of simultaneous speakers is introduced as a regularization process [18].

## 2. State-of-the-Art

Since the overlapped speech signal is a common case in cocktail party scenarios, proposing a method for estimating the number of simultaneous speakers is an important task in these systems. Humans can separate one speaker from a mixture of speech signals due to their natural capacity [19], and they use this benefit to estimate the number of simultaneous speakers in real conditions. In the last decade, a limited range of methods were proposed for speaker counting based on audio signals, where low accuracy in noisy and reverberant scenarios is the main weakness in these algorithms. Kumar et al. proposed an NoS estimating method in 2011 based on the Bessel features of the recorded signal from simultaneous speakers [20]. The presented method is implemented on the recorded signals of multiple active speakers based on a pair of spatially separated microphones. The captured signals of the speech sources are considered for estimating the delay of the speech signals between the source and microphones, which are calculated by a cross-correlation function. The peaks in the cross-correlation function are not trustable due to the noise, reverberation, and sinusoidal components in the recorded speech signal based on the vocal track responses. These undesirable effects are reduced by limiting the components of the speech signal on low frequency bands based on a proper range of the Bessel coefficients. The Bessel function provides proper features of speech signals due to regular zero crossing, which is suitable for the processing of the speech signal. The proposed method estimates the NoS in scenarios where the number of simultaneous speakers is less than the number of microphones, but the accuracy is decreased in the reverberant scenarios. Tomasz and Miroslaw proposed a speaker counting method in 2018 based on human–machine interaction systems [21]. The interreference between different sources significantly reduces the performance of the system in many applications. They proposed a speaker counting method based on the tracking of spectral peaks and extracted features of the spectrum. The statistical properties of extracted features are considered for detecting the relation between speakers, even when the features are not clear in some conditions. In addition, the non-negative matrix factorization method has been selected for classifying the signal spectrum for evaluating the perception of the speech signal. Fabian et al. in 2019 proposed a method for estimating the NoS based on supervised learning [22]. They presented a unifying probabilistic criterion, where the deep neural network is utilized for extracting the output posterior function. These probabilities are evaluated for obtaining discrete point estimations. They showed that convolutional neural networks are a proper instrument for the evaluations based on extracted features of the speech signal. In addition, the presented method is robust in terms of the gain variation of the speech signal for different datasets, noise, and reverberation based on comprehensive evaluations. Humans based on their auditory and perceptual system, focus on a specific speaker in noisy spaces with simultaneous overlapped speakers. Based on this norm, Valentin et al. in 2019 considered the human brain characteristics for proposing a speaker counting system [23]. They designed a perceptual study for evaluating the participant’s capacity for speaker counting in an acoustical environment. This research group considered speech diarization-based models due to the acoustical characteristics of the speech signal for estimating the number of speakers. In addition, the performance of the diarization system is increased for estimating the NoS by reducing the searching space for output sentences. If the proposed method is implemented over long time frames, the probability of detecting simultaneous speakers is increased for each time–frequency (TF) point, which is the benefit of using a limited search space. The presented method has been evaluated on various datasets, which estimate the number of simultaneous speakers, similar to human capacity. Shahab et al. in 2017 proposed a blind speaker counting method in the reverberant conditions based on clustering coherent features [24]. The proposed method utilizes magnitude square coherence in the frequency domain (FD-MSC) between two recorded speech signals of different speakers as a trustable feature for detecting the number of speakers in reverberant environments. It has been shown that speakers have different FD-MSC features, which are obtained from the recorded speech signals of two microphones. The proposed method distinguishes the speakers based on their unique sound features, which are modeled by speech signals. Normally, speaker counting methods require the inter-sensor distance, which is not a requirement in this algorithm. In addition, the presented method is robust in noisy and reverberant scenarios for different inter-sensor distances. Ignacio et al. in 2018 presented a speaker counting method based on the variation of Bayesian probabilistic linear discriminant analysis (PLDA) in diarization techniques [25]. They presented a system based on i-vector PLDA, where the i-vector observations are calculated and extracted for each time point of the speech signal based on an initial segmentation process. These i-vector values are classified based on the full Bayesian PLDA algorithm for the proposed system configuration. This model generates the diarization labels by averaging the repetitive Bayes variables based on the hidden parameters as the speakers’ labels. The number of speakers is estimated by a comparison between various hypotheses based on different information criteria. Pierre et al. in 2020 proposed a speaker counting method with high resolution based on improved convolutional neural networks [26]. In the first step, they introduced multi-channel signals as the input of neural networks in the NoS estimating application. In the proposed method, the ambisonics features of multi-channel microphone arrays are combined with convolutional recurrent neural networks (AF-CRNN). The accuracy of CRNN algorithms is increased for estimating the NoS by extracting the sound signals’ features in short timeframes, which is considered to be proper information for speaker separation and localization algorithms. Junjie et al. in 2019 proposed a speaker counting method based on density clustering and classification decision (SC-DCCD) on overlapped frames of speech signals [27]. In a reverberant scenario, the performance of TF methods is reduced based on the short-times frames in real conditions. To tackle this challenge, a density-based classification method was proposed for estimating the NoS based on a local dominant speaker in the recorded speech signals. The proposed algorithm contains various clustering steps based on a high reliability assumption in the signals’ frames. In the first step, the dominant eigenvectors are calculated by a local covariance matrix of the mixed TF speech components. Then, the eigenvalues are ranked based on the combination between local densities and the distance to the other local eigenvectors with better density. In the second step, a gap-based method is presented for calculating the clusters’ centers of the ranked eigenvectors in the real frequency bins. In the third step, a criterion based on the averaged clusters’ centers is introduced for preparing trustable clusters for the decision step. The presented method provides suitable estimations in reverberant and no-noise conditions.

In this article, a novel method is proposed for speaker counting by a new hive shaped nested microphone array (HNMA) in combination with wavelet packet transform (WPT) for smart sub-band processing and a two-dimensional steered response power (SRP) algorithm with adaptive phase transform (PHAT) and maximum likelihood (ML) weighting functions. Aliasing and imaging are important factors, which affect the frequency component of a speech signal. Therefore, an HNMA by the extension capacity is proposed for eliminating aliasing and imaging. Since the speech signal contains different information in each time frame, the Blackman–Tukey method is proposed for spectral estimation of the speech signal for eliminating the area with low speech components. In addition, the WPT is proposed for smart sub-band processing due to the variable resolution of the speech signal in different frequencies. In the following, the modified SRP algorithm is implemented in two dimensions on the sub-band information of the speech signal. Furthermore, various weighted functions are calculated in each sub-band for considering the effect of the SRP function. In addition, the weighting PHAT and ML functions are adaptively combined with the 2DASRP algorithm. Therefore, the standard deviation (SD) parameter is calculated for each sub-band, and the peak positions of the SB-2DASRP function in this range are passed and the rest eliminated. In the following, the passed peak positions are considered as the input for the unsupervised agglomerative classification method, and the number of clusters (speakers) is estimated based on the elbow criteria. The basic idea of this article was presented in our conference paper [28] for a simple scenario on limited space. Furthermore, the proposed HNMA-SB-2DASRP algorithm is compared with more complex methods such as FD-MSC, AF-CRNN, and SC-DCCD.

Section 3 shows the models for microphone signals in real and simulated conditions and also the HNMA for eliminating aliasing between the microphone signals. Section 4 presents the proposed NoS estimating method based on Blackman–Tukey spectral estimation, sub-band processing by WPT, and the modified two-dimensional sub-band SRP function by adaptive use of PHAT and ML filters in combination with unsupervised agglomerative clustering with elbow criteria. In Section 5, the simulations of the proposed HNMA-SB-2DASRP algorithm are presented and compared to the FD-MSC, i-vector PLDA, AF-CRNN, and SC-DCCD methods in different noisy and reverberant scenarios. Furthermore, the computational complexity of the method is evaluated and compared with other works in this section. Finally, Section 6 includes the conclusions of the proposed system for real-time implementation with different numbers of simultaneous speakers.

## 3. Microphone Models and the Proposed Nested Microphone Array

Speech signal processing algorithms such as estimating the NoS, source localization, and speaker tracking are highly dependent to the quality of the recorded signals. The quality of the signals can affect the accuracy of the algorithms and decrease the reliability of the methods, which prevent them from being implemented in real conditions. The algorithms are first implemented on simulated data. If the obtained results are favorable, the methods are tested and evaluated on real data. In this article, the microphone signal model is introduced and compared for ideal and real acoustical scenarios. Then, the HNMA with the capacity for extension is proposed for data recording and eliminating aliasing.

### 3.1. Microphone Signal Model for Real and Ideal Conditions

**Figure 1:**
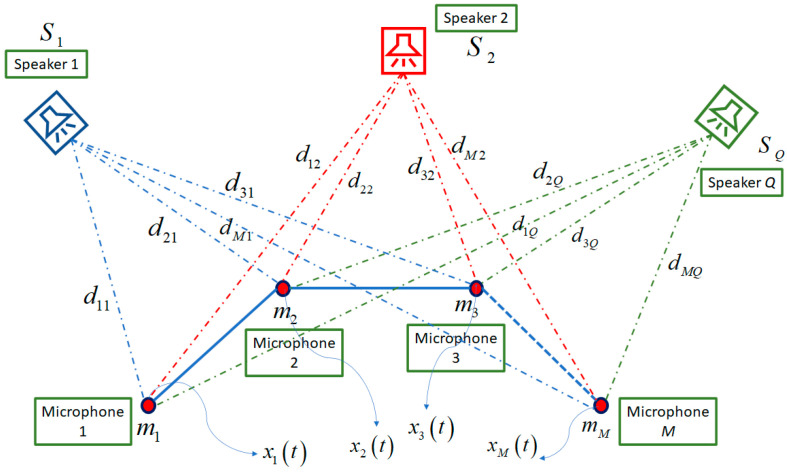
The near-field model for simulating the recorded signals by microphone array.

The recorded signals by microphones are the principal part for all speech processing algorithms. In real conditions, recorded speech should be synchronized to prepare the correct information for delay-based algorithms in signal processing scenarios. Two main assumptions, namely, near-field and far-field, are considered for microphone signals. In the near-field scenario, the distance between the source and the microphones is short in comparison to the array dimension, and the speech waves reach the array in a spherical shape. Instead, in the far-field scenario, the speaker is far from the array, and the waves reach the microphone array parallelly. As shown in Figure 1, the near-field assumption is selected for microphone signals based on the indoor scenario.

In addition, the real and ideal models are considered for the recorded signals based on the environmental scenarios. In the ideal model, it is assumed that delays and amplitude attenuation are observed in the recorded signals as

(1) $$x_{m}\left(t\right)=\sum_{q=1}^{Q}\frac{1}{r_{mq}}S_{q}\left(t-\tau_{mq}\right)+v_{m}\left(t\right)\;\mathrm{for}\;m=1,\ldots ,M,$$

where in Equation (1), $x_{m}\left(t\right)$ is the captured signal by *m*-th microphone based on *Q* sound sources, $S_{q}\left(t\right)$ is the propagated signal by *q*-th source, $r_{mq}$ is the distance between the *m*-th microphone and the *q*-th source, $\tau_{mq}$ is the time delay between the *q*-th source and the *m*-th microphone, $v_{m}\left(t\right)$ is the additive Gaussian noise in the *m*-th microphone place, *M* is the number of microphones, and *Q* is the number of sound sources. This model is named ideal because of not having the effect of reverberation, but as is known, environmental reflection is an important challenge of the recorded signals in indoor scenarios. Therefore, the real model is proposed to prepare the simulated signal similar to real recording speech as

(2) $$x_{m}\left(t\right)=\sum_{q=1}^{Q}S_{q}\left(t\right)*\xi \left(d_{mq},t\right)+v_{m}\left(t\right)\;\mathrm{for}\;m=1,\ldots ,M,$$

where in Equation (2), $\xi \left(d_{mq},t\right)$ is the room impulse response between the *q*-th source and the *m*-th microphone, and * is the convolution operator. This room impulse response includes the amplitude attenuation, the room reverberation effects, and the time delays between the source and microphones. By considering the real model, simulated signals in the microphone array are highly similar to the recorded signals.

### 3.2. The Proposed Hive Shaped Nested Microphone Array

**Figure 2:**
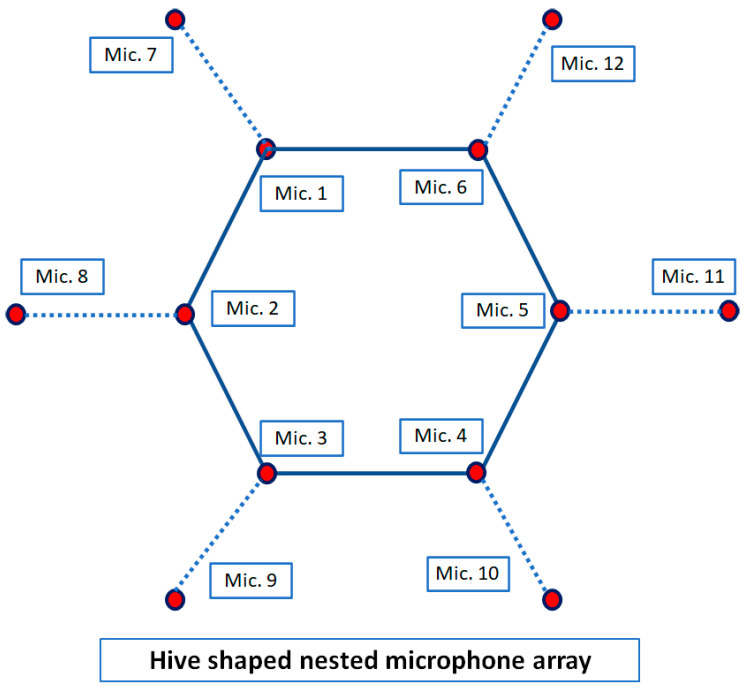
The structure of the proposed hive-shaped nested microphone array (HNMA) for estimating the number of speakers.

Noise and reverberation are undesirable environmental factors, which affect the quality of the signals, and they are highly controlled and eliminated by using signal processing algorithms. Aliasing is another factor that affects the frequency components of the speech signal, which happens based on inter-sensor distances. In recent years, research has been proposed for eliminating the aliasing. For example, in [29], a linear nested microphone array was presented for eliminating aliasing in speech enhancement applications. In this article, an HNMA with extension capacity is proposed for eliminating aliasing, which is shown in Figure 2. As seen, the proposed HNMA is designed with spatial symmetry, which provides identical speech recording from all speakers. In addition, based on the special hive shape in the array, it provides the capacity for cell extensions in the required scenarios. In the following, a strategy to design an HNMA is explained together with the related analysis filters.

**Figure 3:**
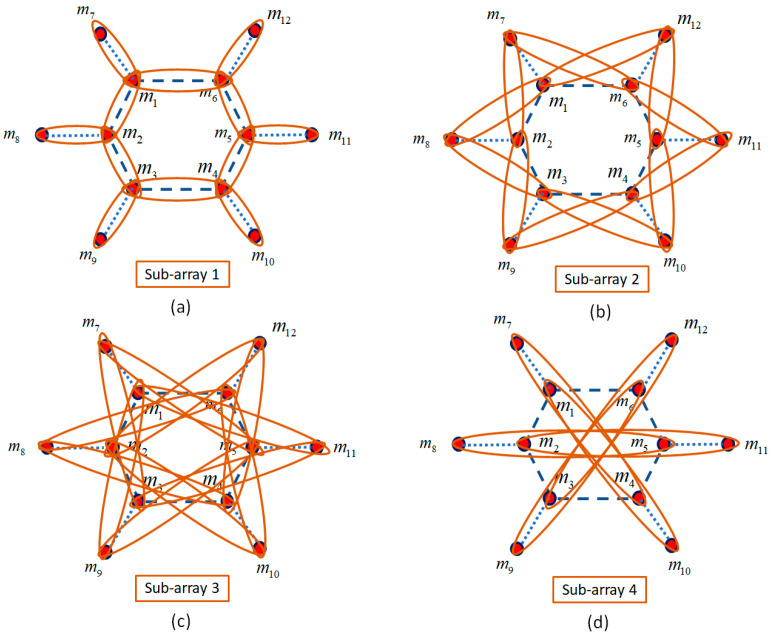
The microphone pairs related to the proposed HNMA for the (**a**) first sub-array, (**b**) second sub-array, (**c**) third sub-array, and (**d**) fourth sub-array.

The proposed HNMA is designed to use a set of microphones for each sub-array together with analysis filter banks and down samplers, where each sub-array is structured for a specific frequency range. The speech signal with the frequency range B = [0–7800] Hz by sampling frequency $F_{s}=16000\mathrm{Hz}$ is considered for the speech processing algorithms. Therefore, the proposed HNMA should be designed in such a way to cover the speech signal components in this frequency range. The Nyquist theorem (d≺ϒ/2) is considered for avoiding the aliasing and imaging, where *d* is the inter-sensor distance, and ϒ is the highest frequency of the signal in each frequency band. Therefore, the first sub-array is designed to cover the frequency range B1 = [3900–7800] Hz, where in this condition, the inter-sensor distance and central frequency are $d_{1}<2.15\mathrm{cm}$ and $fd_{1}=5850\mathrm{Hz}$, respectively. The second sub-array should cover the frequency period B2 = [1950–3900] Hz, where for this sub-array, the inter-sensor distance is $d_{2}=2d_{1}<4.3\mathrm{cm}$, and the central frequency is $fd_{2}=2925\mathrm{Hz}$. The third sub-array is structured for the frequency range B3 = [975–1950] Hz. The conditions for this sub-array to be implemented are $d_{3}=4d_{1}<8.6\mathrm{cm}$ as the inter-sensor distance and $fd_{3}=1462\mathrm{Hz}$ as the central frequency for the analysis filter. Finally, the fourth sub-array is designed to cover the frequencies B4 = [0–975] Hz, and similar to other steps, the inter-sensor distance and central frequency for the analysis filter are $d_{4}=8d_{1}<17.2\mathrm{cm}$ and $fd_{4}=487\mathrm{Hz}$, respectively. The proposed HNMA includes 12 microphones, which are placed in a specific shape for preparing the characteristics of the nested structure. Figure 3 shows four sub-arrays related to each frequency range. 

As shown in Figure 3, each sub-array is considered with the related microphone pairs, which prepares the information for the NoS estimating algorithm. The first sub-array, which covers the highest frequency range, includes the microphone pairs (1,2), (2,3), (3,4), (4,5), (5,6), (6,1), (1,7), (2,8), (3,9) (4,10), (5,11), and (6,12), where the inter-sensors distance in this sub-array is $d_{1}=2.15\mathrm{cm}$. The microphone pairs (8,1), (8,3), (9,2), (9,4), (10,3), (10,5), (11,4), (11,6), (12,5), (12,1), (7,6), and (7,2) are considered for the second sub-array with inter-sensor distance $d_{2}=4.1\mathrm{cm}$. The HNMA is designed to consider the microphone pairs (8,6), (8,4), (9,1), (9,5), (10,2), (10,6), (11,3), (11,1), (12,4), (12,2), (7,5), and (7,3) for the third sub-array with inter-sensor distance $d_{3}=7.35\mathrm{cm}$. Finally, the microphone pairs (7,4), (10,1), (8,5), (2,11), (9,6), and (3,12) are selected for the fourth sub-array with $d_{4}=9.85\mathrm{cm}$ to prepare the proper information for the lowest frequency range of the speech signal.

**Figure 4:**
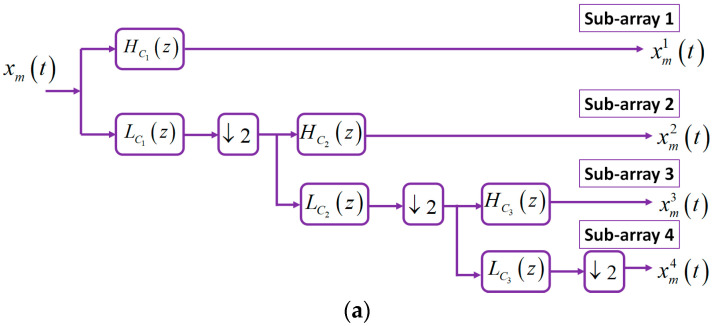

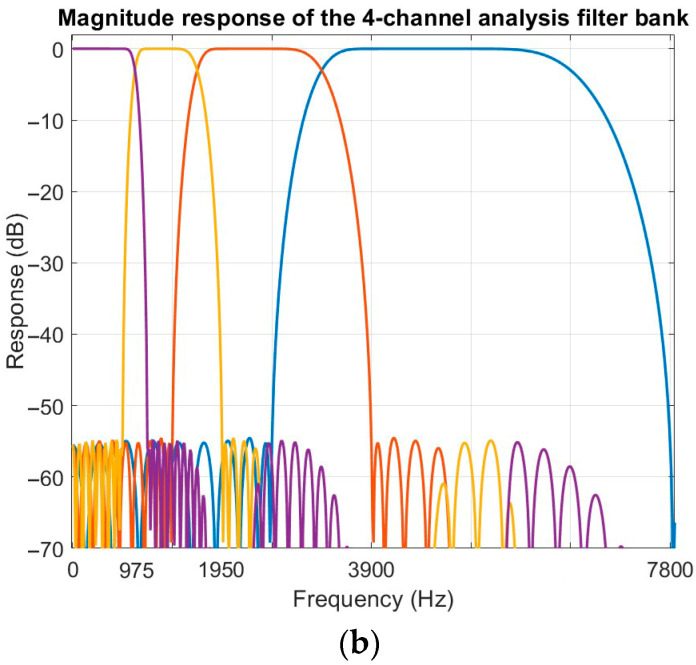
(**a**) A tree structure for implementing the analysis filter bank, and (**b**) the frequency spectrum related to the analysis filter for each sub-array of the HNMA.

A proper analysis filter bank is required for each sub-band to avoid aliasing and imaging. As seen in Figure 4a, an analysis filter $H_{i}\left(z\right)$ together with a down sampler $C_{i}$ are implemented in a tree structure. Each level of the tree includes a high-pass filter (HPF) $H_{C_{i}}\left(z\right)$, a low-pass filter (LPF) $L_{C_{i}}\left(z\right)$, and a down sampler $C_{i}$. The relation between HPFs, LPFs, and the analysis filters is written as

(3) $$\begin{array}{l} H_{1}\left(z\right)=H_{C_{1}}\left(z\right)\\ H_{2}\left(z\right)=L_{C_{1}}\left(z\right)*H_{C_{2}}\left(z^{2}\right)\\ H_{3}\left(z\right)=L_{C_{1}}\left(z\right)*L_{C_{2}}\left(z^{2}\right)*H_{C_{3}}\left(z^{4}\right)\\ H_{4}\left(z\right)=L_{C_{1}}\left(z\right)*L_{C_{2}}\left(z^{2}\right)*L_{C_{3}}\left(z^{4}\right) \end{array}$$

For implementing the tree structure, a 54-tap HPF and a 54-tap LPF are selected based on the finite impulse response model of the filters, which are designed by the elliptic method. The designed analysis filters contain the stop band 55 dB and transition band 0.0538, which provide accurate filters for frequency component separation. Figure 4b represents the frequency spectrum related to the analysis filter for each sub-array of the proposed HNMA. The analysis filter $H_{1}\left(z\right)$ is considered for the sub-array with the shortest inter-sensor distance, and the analysis filter $H_{4}\left(z\right)$ is for the sub-array with longest inter-microphone distance. In practice, aliasing is eliminated between the microphone pairs by using these analysis filters and down samplers together with the special structure of the proposed HNMA.

## 4. The Proposed Speaker Counting Method Based on WPT and Sub-Band-2DASRP Algorithm

**Figure 5:**
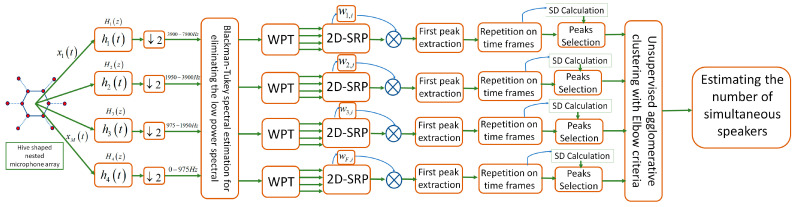
The block diagram of the proposed NoS estimating system based on Blackman–Tukey spectral estimation, WPT, and SB-2DASRP by agglomerative classification and elbow criteria.

The proposed NoS estimating method is based on a novel HNMA in combination with Blackman–Tukey spectral estimation for detecting the frequency areas with proper components and eliminating the undesirable areas with low speech signals. Figure 5 shows the block diagram of the proposed method for speaker counting in real acoustical environments. In the following, the WPT method is presented for smart sub-band signal processing with special attention to the frequency components of each sub-array. In addition, the SRP algorithm, as a principal method, is implemented on sub-bands with a modification in 2D conditions and adaptively by weighting PHAT and ML filters on each sub-array. Some weighted factors are introduced for each sub-band to highlight the effect of dominant speakers for extracting the proper peaks positions. Finally, the unsupervised agglomerative clustering method is implemented on the extracted data, and the elbow criteria is selected for estimating the number of simultaneous speakers. Figure 5 shows each part of the algorithm separately, which are explained in the next section.

### 4.1. The Blackman–Tukey Spectral Estimation for Multi-Speaker Speech Signal

Speech is a non-stationary signal, where each time frame contains the different frequency components with variable and non-uniform distributions. In some frames, the low frequency components are highlighted due to the speaking of vowels, and in other frames, the high frequency components are dominant because of consonants. However, in both conditions, the noise signal is always dominant in the frames with low speech components. Therefore, Blackman–Tukey estimation [30] is proposed as a proper algorithm for speech spectral estimation and to eliminate the frames with low frequency components of the speech and high frequency components of the noise. The advantage of the Blackman–Tukey method is providing a smooth spectrum of the speech signal, where the undesirable spectral components are eliminated by selecting a proper spectral threshold. This threshold is selected by experiment to obtain a balance between computational complexity and accuracy. 

The Blackman–Tukey spectral estimation method is an enhanced version of a periodogram, which decreases the spectral estimation variances by implementing the windows on the signal. If we have *N* samples of signal $x\left[n\right]\;\left(n=0,\ldots ,N-1\right)$, the discrete Fourier transform of signal $x\left[n\right]$ is expressed as

(4) $$X\left[k\right]=\Delta T\sum_{n=0}^{N-1}x\left[n\right]e^{-j2\pi \frac{kn}{N}}\;\;k=0,\ldots ,N-1,$$

where in Equation (4), ΔT is the sampling period ($\Delta T=\frac{1}{f_{s}}$, and $f_{s}$ is the sampling frequency). The spectrum estimation range in the frequency domain is selected as

(5) $$-\frac{\pi }{\Delta T}\leq \omega \leq \frac{\pi }{\Delta T}.$$

Based on the definition of spectral estimation, the speech spectrum is expressed as

(6) $${\hat{\Gamma }}_{xx}\left[k\right]=\Delta T\sum_{m=-M}^{M}{\hat{\gamma }}_{xx}\left[m\right]e^{-j\omega m\Delta T}\;\;k=0,1,\ldots ,N-1$$

where in Equation (6), ${\hat{\gamma }}_{xx}\left[m\right]$ for m=−M,…,M is the discrete estimation of the autocorrelated function, which is normally considered to be composed of biased and unbiased estimators. For the unbiased estimators, the discrete estimators are written as

(7) $${\hat{\gamma }}_{xx}\left[m\right]=\frac{1}{N-m}\sum_{n=0}^{N-m-1}x\left[n\right]x\left[n+m\right]\;\;m=0,1,\ldots ,N-1,$$

and for biased estimators, these estimations are

(8) $${\hat{\gamma }}_{xx}\left[m\right]=\frac{1}{N}\sum_{n=0}^{N-m-1}x\left[n\right]x\left[n+m\right]\;\;m=0,1,\ldots ,N-1,$$

where finally the expected value of the biased estimators from the autocorrelation function is summarized as

(9) $$E\left\{{\hat{\gamma }}_{xx}\left[m\right]\right\}=\frac{N-m}{N}\gamma_{xx}\left[m\right],$$

where it is a real autocorrelation function, which is weighted by a triangular window. The biased estimator (Equation (8)) contains less mean square error in comparison to the unbiased estimator (Equation (7)), which makes it a proper estimator for many applications in speech processing. In the Blackman–Tukey method, the biased estimator (Equation (8)) is considered in Equation (6), which is selected for estimating the autocorrelation function ${\hat{\gamma }}_{xx}\left[m\right]$ of the speech signal. Therefore, Equation (6) can be solved by averaging fast Fourier transform values. In practice, the autocorrelation ${\hat{\gamma }}_{xx}\left[m\right]$ is zero padded for preparing a smooth spectrum. The smoothing level of the Blackman–Tukey estimator is dependent on the correlated coefficients and the number of zero padded values, which are selected by experiment to obtain a proper spectrum of the speech signal.

### 4.2. Smart Sub-Band Processing for Speech Signal by Wavelet Packet Transform

In this section, the sub-band processing step is proposed to focus on the variable frequency components of the speech signal. The speech is a wideband and non-stationary signal, which means there are variable components in different frequency bands and time frames. In addition, the speech is a signal with windowed-disjoint orthogonality properties with high probability to have just one active speaker in each TF bin of the overlapped speech signal [31]. Since signal processing on each TF bin considers high computational complexity, we propose smart sub-band processing with WPT [32], which provides a benefit to having various frequency resolutions in different TF bands of the speech signal. The continues wavelet transform (CWT) is used for extracting the translation and scale parameters of a continues signal. If the recorded and enhanced speech signal by the HNMA and Blackman–Tukey method is considered as ${\hat{x}}_{i}\left(t\right)$, the CWT of the signal is defined as

(10) $${\hat{W}}_{\Psi }\left(s,\tau \right)=\int_{-\infty }^{+\infty }{\hat{x}}_{i}\left(t\right)\psi_{s,\tau }^{*}\left(t\right)dt,$$

where in Equation (10), ${\hat{W}}_{\Psi }\left(s,\tau \right)$ is the CWT, and $\psi_{s,\tau }^{}\left(t\right)$ is expressed as

(11) $$\psi_{s,\tau }^{}\left(t\right)=\frac{1}{\sqrt{s}}\psi \left(\frac{t-\tau }{s}\right),$$

where in Equation (11), *s* and τ are known as the scale and translation parameters, respectively. The discrete wavelet transform (DWT) [33] is proposed for implementing the wavelet transform on discrete-time signals, which is similar to CWT with scale and translation as discrete parameters $\tau =k\times 2^{J}$ and $S=2^{J}$, where $\left\{J,k\right\}\in {\mathbb{Z}}^{2}$. Therefore, the DWT is defined as

(12) $$\psi_{s,\tau }^{}\left(t\right)=\frac{1}{\sqrt{2^{J}}}\psi \left(\frac{t-k\times 2^{J}}{2^{J}}\right)$$

**Figure 6:**
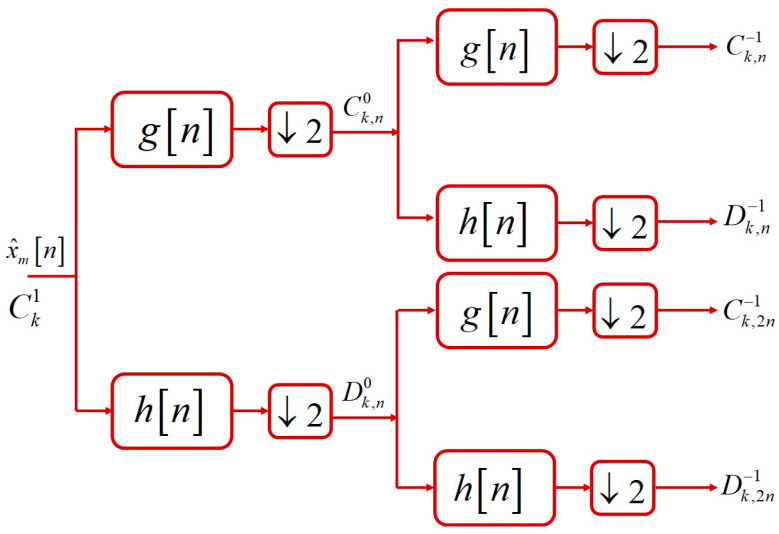
The 2-level decomposition of 2-channel WPT for the speech signal based on a recursive filter bank.

The signal decomposition is the main challenge in DWT implementations, where the common method is the combination with HPFs, LPFs, and down samplers. The numbers of channels and decomposition levels are dependent on the application and the considered signal in the simulation scenarios. The WPT is an extension of DWT for having better performance in different frequency ranges. The benefit of WPT is that it not only has different frequency resolutions at low frequencies, but it also prepares variable resolutions in high frequencies. The WPT, in comparison to DWT performs better for audio and speech signals. The WPT is considered for the proposed NoS estimating system, which provides 4 sub-bands for each sub-array, where finally there are 16 sub-bands for all frequency ranges of the speech signal. Figure 6 shows the implementation of 2-channel and 2-level WPTs on the recorded signal by HNMA based on a recursive filter bank. The details of WPT implementation are explained in the following.

In the decomposition step of WPT implementation, the low-pass and high-pass bands are equally divided into two sub-bands. This process is frequently repeated in low and high frequencies for preparing the proper frequency resolution of the speech signal. In DWT, the low frequency components of the signal are divided into each level of decomposition, but in WPT not only the low frequencies but also the high frequencies are divided into smaller sub-bands. If the wavelet coefficients for enhanced sub-array signals ${\hat{x}}_{i}\left(t\right)$ are considered as $C_{k}^{J+1}$, in each decomposition level of WPT, the detail signal ($D_{k}^{J}$) and coarse signal ($C_{k}^{J}$) are calculated, respectively, as

(13) $$\begin{array}{l} C_{k}^{J}=2^{-\frac{J}{2}}\int_{-\infty }^{+\infty }{\hat{x}}_{i}\left(t\right)\bar{\psi }\left(2^{-J}t-k\right)dt\\ D_{k}^{J}=2^{-\frac{J}{2}}\int_{-\infty }^{+\infty }{\hat{x}}_{i}\left(t\right)\bar{\varphi }\left(2^{-J}t-k\right)dt \end{array}$$

where in Equation (13), $\varphi \left(t\right)$ is the scale function, and $\psi \left(t\right)$ is the wavelet function for implementing the speech signal, which are expressed as

(14) $$\begin{array}{l} \psi (t)=\sum_{n}g\left[n\right]\sqrt{2}\varphi (2t-n)\\ \varphi (t)=\sum_{n}h\left[n\right]\sqrt{2}\varphi (2t-n) \end{array}$$

where in Equation (14), $g\left[n\right]$ is an HPF, and $h\left[n\right]$ is an LPF in each level of the WPT implementation. The first LPF and HPF in the WPT structure are considered for the frequency range [0–3900] Hz and [3900–7800] Hz, respectively. The relation between LPF and HPF for recursive implementation is expressed as

(15) $$g\left[n\right]={\left(-1\right)}^{n}h\left[D+1-n\right]\;\;n=1,\ldots ,D,$$

where in Equation (15), *D* is the filter length. In a recursive implementation, the LPF is generated by the elliptic method as a finite impulse response filter, and the HPF is calculated by using Equation (15) based on a mathematical equation. If the WPT coefficient for the reference signal is considered to $C_{k}^{1}$, the coarse signal ($C_{k,n}^{0}$) and detail signal ($D_{k,n}^{0}$) are calculated by using Equation (13) in the first level of decomposition. In the second level of WPT, the coarse signal ($C_{k,n}^{-1}$) and detail signal ($D_{k,n}^{-1}$) are calculated based on Equation (14) for high frequencies as

(16) $$\begin{array}{l} C_{k,n}^{-1}=\sum_{l}g\left[l-2k\right]C_{k,n}^{0}\\ D_{k,n}^{-1}=\sum_{l}h\left[l-2k\right]C_{k,n}^{0} \end{array}$$

In a similar process, the coarse signal ($C_{k,2n}^{-1}$) and detail signal ($D_{k,2n}^{-1}$) are calculated for the low frequencies, respectively, as

(17) $$\begin{array}{l} C_{k,2n}^{-1}=\sum_{l}g\left[l-2k\right]D_{k,n}^{0}\\ D_{k,2n}^{-1}=\sum_{l}h\left[l-2k\right]D_{k,n}^{0} \end{array}$$

Therefore, the high and low frequency bands are equally divided into two other sub-bands in each decomposition level of WPT implementation. Then, the high frequency resolution is proposed by WPT for sub-arrays of HNMA related to low frequency components. Furthermore, a lower frequency resolution is produced for high frequency components of the sub-arrays but with the same implementation of WPT. Therefore, the output of each level of WPT for microphone signals from HNMA is expressed as

(18) $${\hat{x}}_{i,f}\left[n\right]={\hat{x}}_{i}\left[n\right]*h_{f}\left[n\right]\;\;for\;f=1,\ldots ,F,$$

where in Equation (18), $h_{f}\left[n\right]$ is the equivalent WPT filter for each level of sub-band processing on the microphone signals of HNMA, and *F* is the number of sub-bands. Various mother wavelets have been introduced for implementing digital signals such as Haar, Biorthogonal, Coiflet, Morlet, Mexican Hat, Daubechies, etc., where the Daubechies mother wavelet is selected for implementing the speech signal due to its high performance in comparison to other wavelets. In the next section, the modified sub-band 2D-adaptive SRP function is introduced for implementing the obtained sub-band signals of WPT.

### 4.3. The Sub-Band 2D-Adaptive SRP Implementation with PHAT and ML Weighting Functions

Different categories of the SSL methods have been proposed in recent years. One group of methods is based on environmental energy calculation, and another category is implemented by using time difference of arrival estimation. In this article, a novel system for estimating the NoS is proposed based on an enhanced version of the localization method and validating the obtained results by unsupervised clustering and classification algorithms. A modified 2D version of the SRP [34] method is adaptively considered for implementing the novel NoS estimating system by PHAT and ML weighting functions. The microphone signal model in Equation (2) clearly shows the existence of the filtered and delayed version of the source signal $S\left(t\right)$ in each microphone for an *M*-microphone array. The delay-and-sum beamformer and filter-and-sum beamformer (FSB) methods are the common assumptions for microphone array signal processing.

Since the reverberation contains the non-line-of-sight version of the source signal, the noise $v\left(t\right)$ is highly correlated with the source signals $S\left(t\right)$. Therefore, the delay-and-sum beamformer is not effective in these conditions. The FSB method solves the correlation challenge by implementing adaptive filters on the microphone signals. The output of the FSB in the frequency domain for the *f*-th sub-band with *M* microphones is expressed as

(19) $$P_{f}\left(\omega ,\Delta_{1},\ldots ,\Delta_{M}\right)=\sum_{m=1}^{M}G_{m,f}\left(\omega \right){\hat{X}}_{m,f}^{}\left(\omega \right)e^{-j\omega \Delta_{m}},$$

where in Equation (19), ${\hat{X}}_{1,f}^{}\left(\omega \right),\ldots ,{\hat{X}}_{M,f}^{}\left(\omega \right)$ are the Fourier transform of the enhanced microphone signals for different sub-bands, and $G_{1,f}^{}\left(\omega \right),\ldots ,G_{M,f}^{}\left(\omega \right)$ are the Fourier transform of the adaptive filters in the FSB structure. The steered response is a function of *M* steered delays $\Delta_{1},\ldots ,\Delta_{M}$ in the acoustical environment. The SRP for the *f*-th sub-band based on the FSB structure is obtained as

(20) $$Y_{f}^{SB}\left(\Delta_{1},\ldots ,\Delta_{M}\right)=\int_{-\infty }^{+\infty }P_{f}\left(\omega ,\Delta_{1},\ldots ,\Delta_{M}\right){P^{\prime }}_{f}\left(\omega ,\Delta_{1},\ldots ,\Delta_{M}\right)d\omega$$

where $P_{f}\left(\omega ,\Delta_{1},\ldots ,\Delta_{M}\right)$ is the FSB output for the *f*-th sub-band, and ${P^{\prime }}_{f}\left(\omega ,\Delta_{1},\ldots ,\Delta_{M}\right)$ is its complex conjugate. The SRP function is implemented on a 2D area as a modified version of the energy-based algorithms. For implementation, the Z direction is considered as a fixed value based on the average height of the people inside of the room, and the SB-SRP function is implemented on X and Y dimensions based on the selected two dimensions. In practice, the SRP function is expressed as the average of microphone pairs, which is equivalent to the summation of generalized cross-correlation of all microphone pairs as

(21) $$Y_{f}^{SB-2D}\left(\Delta_{1},\ldots ,\Delta_{M}\right)=\int_{-\infty }^{+\infty }\left(\sum_{a=1}^{M}G_{a,f}\left(\omega \right){\hat{X}}_{a,f}\left(\omega \right)e^{-j\omega \Delta_{a}}\right)\left(\sum_{b=1}^{M}{G^{\prime }}_{b,f}\left(\omega \right){{\hat{X}}^{\prime }}_{b,f}\left(\omega \right)e^{j\omega \Delta_{b}}\right)d\omega$$

By merging the sigma operators, Equation (21) is simplified as

(22) $$Y_{f}^{SB-2D}\left(\Delta_{1},\ldots ,\Delta_{M}\right)=\int_{-\infty }^{+\infty }\sum_{a=1}^{M}\sum_{b=1}^{M}\left(G_{a,f}\left(\omega \right){\hat{X}}_{a,f}\left(\omega \right)e^{-j\omega \Delta_{a}}\right)\left({G^{\prime }}_{b,f}\left(\omega \right){{\hat{X}}^{\prime }}_{b,f}\left(\omega \right)e^{j\omega \Delta_{b}}\right)d\omega$$

For more simplification based on the variables in Equation (22), the sigma and integral are replaced as

(23) $$Y_{f}^{SB-2D}\left(\Delta_{1},\ldots ,\Delta_{M}\right)=\sum_{a=1}^{M}\sum_{b=1}^{M}\int_{-\infty }^{+\infty }G_{a,f}\left(\omega \right){G^{\prime }}_{b,f}\left(\omega \right){\hat{X}}_{a,f}\left(\omega \right){{\hat{X}}^{\prime }}_{b,f}\left(\omega \right)e^{j\omega \left(\Delta_{b}-\Delta_{a}\right)}d\omega ,$$

where in Equation (23), the weighting function is defined as

(24) $$\Omega_{ab,f}\left(\omega \right)=G_{a,f}\left(\omega \right){G^{\prime }}_{b,f}\left(\omega \right).$$

If the weighting function is replaced in Equation (23), the SB-2DSRP function is expressed as

(25) $$Y_{f}^{SB-2D}\left(\Delta_{1},\ldots ,\Delta_{M}\right)=\sum_{a=1}^{M}\sum_{b=1}^{M}\int_{-\infty }^{+\infty }\Omega_{ab,f}\left(\omega \right){\hat{X}}_{a,f}\left(\omega \right){{\hat{X}}^{\prime }}_{b,f}\left(\omega \right)e^{j\omega \left(\Delta_{b}-\Delta_{a}\right)}d\omega .$$

The SRP method without specific weighting functions has low performance in noisy and reverberant scenarios. Therefore, the adaptive use of weighting filters is proposed in the SRP function for increasing the performance of the algorithm. In [35], it was shown that the PHAT filter works better in reverberant scenarios, and the ML function has better performance in noisy conditions. Therefore, the adaptive use of these filters is proposed in combination with the SRP function by estimating the level of the noise and reverberation in the environment. The PHAT filter is considered for whitening the cross spectrum of the microphone signals, which is defined as

(26) $$\Omega_{ab,f}^{PHAT}\left(\omega \right)=\frac{1}{\left|{\hat{X}}_{a,f}\left(\omega \right){{\hat{X}}^{\prime }}_{b,f}\left(\omega \right)\right|}$$

If the PHAT filter is considered in the SRP function, the sub-band-2DSRP (PHAT) is expressed as

(27) $$Y_{f}^{SB-2D-PHAT}\left(\Delta_{1},\ldots ,\Delta_{M}\right)=\sum_{a=1}^{M}\sum_{b=1}^{M}\int_{-\infty }^{+\infty }\frac{1}{\left|{\hat{X}}_{a,f}\left(\omega \right){{\hat{X}}^{\prime }}_{b,f}\left(\omega \right)\right|}{\hat{X}}_{a,f}\left(\omega \right){{\hat{X}}^{\prime }}_{a,f}\left(\omega \right)e^{j\omega \left(\Delta_{b}-\Delta_{a}\right)}d\omega$$

By low multi-directional propagation and uncorrelated noise and signals, the ML filter is an effective and unbiased estimator. The ML filter is defined as a function of power spectral density between source signal $S_{q}\left(t\right)$ and noise signals $v_{a}\left(t\right)$ and $v_{b}\left(t\right)$ as

(28) $$\Omega_{ab,f}^{ML}\left(\omega \right)=\frac{\left|S_{q,f}\left(\omega \right)\right|}{\left|v_{a,f}\left(\omega \right)\right|\left|v_{b,f}\left(\omega \right)\right|}{\left\{1+\frac{\left|S_{q,f}\left(\omega \right)\right|}{\left|v_{a,f}\left(\omega \right)\right|}+\frac{\left|S_{q,f}\left(\omega \right)\right|}{\left|v_{b,f}\left(\omega \right)\right|}\right\}}^{2}$$

The ML function is rarely available in real conditions because of the existence the source signal in the formulation. An approximation of the ML filter is expressed as a function of the absolute value of the signal and noise power spectral density, which has high performance on short-time frames of the speech signal as

(29) $${\hat{\Omega }}_{ab,f}^{ML}\left(\omega \right)=\frac{\left|{\hat{X}}_{a,f}\left(\omega \right)\right|\left|{\hat{X}}_{b,f}\left(\omega \right)\right|}{{\left|v_{a,f}\left(\omega \right)\right|}^{2}{\left|{\hat{X}}_{b,f}\left(\omega \right)\right|}^{2}+{\left|v_{b,f}\left(\omega \right)\right|}^{2}{\left|{\hat{X}}_{a,f}\left(\omega \right)\right|}^{2}},$$

where in Equation (29), ${\left|v_{a,f}\left(\omega \right)\right|}^{2}$ and ${\left|v_{b,f}\left(\omega \right)\right|}^{2}$ are the noise power spectrum for microphones $\left\{a,b\right\}$ on different sub-bands, which are estimated from the silence part of the speech signal. Therefore, the SB-2DSRP function is combined with the ML filter, which is expressed as

(30) $$\begin{array}{l} Y_{f}^{SB-2D-ML}\left(\Delta_{1},\ldots ,\Delta_{M}\right)=\\ \sum_{a=1}^{M}\sum_{b=1}^{M}\int_{-\infty }^{+\infty }\frac{\left|{\hat{X}}_{a,f}\left(\omega \right)\right|\left|{\hat{X}}_{b,f}\left(\omega \right)\right|}{{\left|v_{a,f}\left(\omega \right)\right|}^{2}{\left|{\hat{X}}_{b,f}\left(\omega \right)\right|}^{2}+{\left|v_{b,f}\left(\omega \right)\right|}^{2}{\left|{\hat{X}}_{a,f}\left(\omega \right)\right|}^{2}}{\hat{X}}_{a,f}\left(\omega \right){{\hat{X}}^{\prime }}_{a,f}\left(\omega \right)e^{j\omega \left(\Delta_{b}-\Delta_{a}\right)}d\omega \end{array}$$

The peaks positions of the SRP function should theoretically estimate the speakers’ locations or the number of simultaneous speakers. However, the simple result of this algorithm is not valid due to the effects of the environmental noise and reverberation. Therefore, some additional signal processing steps are proposed to reach acceptable results. Firstly, the SB-2DASRP function is implemented on various time frames to obtain the sufficient information. Based on the sparse characteristic of the speech signal, with high probability, where only one speaker is active in overlapped signal frames. Therefore, some weights are defined to highlight the effect of each speaker in the related sub-band. The weights are based on the maximum value of the SB-2DASRP function in each sub-band, which is expressed as

(31) $$w_{f,i}=\frac{\arg\max\left(Y_{f}^{SB-2D-(PHAT/ML)}\left(\Delta_{1},\ldots ,\Delta_{M}\right)\right)}{\sum_{j=2}^{N}\overset{j}{\arg\max}\left(Y_{f}^{SB-2D-(PHAT/ML)}\left(\Delta_{1},\ldots ,\Delta_{M}\right)\right)}\;for\;i=1,\ldots ,N\;\mathrm{and}\;f=1,\ldots ,F$$

where in Equation (31), $\overset{j}{\arg\max}$ is the *j*-th peak position of the SB-2DASRP (PHAT/ML) function in the *f*-th sub-band. Therefore, the peaks of the SB-2DSRP (PHAT/ML) function are multiplied by the proposed weights. These weights highlight the effect of important peaks and reduce the effect of unrelated ones. In the next step after implementing the weights, the biggest value of the SB-2DASRP (PHAT/ML) function is extracted for each sub-band as

(32) $${\hat{T}}_{f}=\arg\max\left(w_{f,i}\times Y_{f}^{SB-2D-(PHAT/ML)}\left(\Delta_{1},\ldots ,\Delta_{M}\right)\right)\;for\;f=1,\ldots ,F,$$

which practically shows the most outstanding source location in each sub-band. This process is repeated for various time frames to extract the related peak positions for various active sources. There are peaks in some frequency bands that are not related to any active speaker, and they appear by the effect of reverberation, where they reduce the accuracy of the final results of the NoS estimating method. Therefore, an SD factor is considered to be a preselection step for selecting the proper peaks and eliminating the undesirable data as

(33) $${\hat{A}}_{i}=\left\{{\hat{T}}_{1},{\hat{T}}_{2},\ldots ,{\hat{T}}_{F}\left|\forall f\in F,\left|{\hat{T}}_{i}\right|>\sigma_{i}\right.\right\},$$

where in Equation (33), $\sigma_{i}$ is the SD for each frequency band, which is calculated as

(34) $$\sigma_{i}=\sqrt{\frac{1}{N}\sum_{j=1}^{N}{\left({\hat{T}}_{j}-\mu \right)}^{2}}\;\mathrm{where}\;\mu =\frac{1}{N}\sum_{j=1}^{N}{\hat{T}}_{j}$$

Therefore, the high-quality peaks related to the real speakers are passed, and the rest of the information is eliminated that does not corresponding to any speaker. Finally, the set of ${\hat{A}}_{i}$ information comprises the candidate points for estimating the NoS, which is considered as the input data for the unsupervised agglomerative classification method with elbow criteria. The mentioned processing step provides trustable information for the classification step to obtain the final correct number of simultaneous speakers with less error.

### 4.4. The Unsupervised Agglomerative Classification with Elbow Criteria

The last step of the proposed system in Figure 5 is the classification of the peak positions from the SB-2DASRP (PHAT/ML) function based on SD preselection. Two categories of classifiers are normally used for data classification as supervised as unsupervised methods. In the supervised classification, the number of clusters is known, and the data are linked to the related class, but in the unsupervised methods, the number of clusters is unknown. Since the number of clusters (speakers) is not clear in the proposed system, an agglomerative classification method [36] is selected for data classifying in the presented system. In addition, the elbow criteria [37] are considered in combination with the classification method for data validation and selecting the optimal number of clusters (speakers). The *N* data are obtained from the last step (${\hat{A}}_{i}$), where ${\vec{Z}}_{i}$ shows the eigenvector of data *i*. The first step in unsupervised agglomerative classification is organizing and grouping the data for preparing the candidate clusters based on a specific metric, which is known as the linkage step. The distance between clusters *V* and *W* is defined as $L\left(W,V\right)$, where different metrics are considered for this distance calculation. Euclidian distance is selected for calculating the inter-cluster distance in the proposed system. In the first step, each cluster contains just one datapoint, which means the number of clusters is equal to the amount of data. In each repetition step, a pair of clusters is evaluated that was not grouped in the last step. Therefore, the clusters with minimum distance *L* are combined to provide the new cluster *U*. If we consider *e* and *r* as the jointed clusters, then $z\left[i\right]$ is defined as data *i* in cluster *z*. After joining the clusters, the distance $L\left(U,V\right)$ is recalculated between new cluster *U* and all other clusters. Various methods were proposed for this distance calculation with a significant effect on the classification results, such as single linkage, average linkage, complete linkage, median linkage, and ward linkage. The ward linkage method is selected in the proposed system for setting the new distance between clusters, which minimizes the within-cluster variance by paring the proper clusters as

(35) $$L\left(U,V\right)=\sqrt{\frac{\left|V\right|+\left|e\right|}{T}L{\left(V,e\right)}^{2}+\frac{\left|V\right|+\left|r\right|}{T}L{\left(V,r\right)}^{2}+\frac{\left|V\right|}{T}L{\left(e,r\right)}^{2}}$$

where in Equation (35), *T* is equal to $\left|V\right|+\left|e\right|+\left|r\right|$, and the ward linkage method is implemented by the nearest-neighbor algorithm. One of the important challenges in calculating the number of clusters and the number of repetitions for classification is inter-cluster distance for merging the similar clusters. Therefore, the elbow criteria are proposed for calculating the optimal number of clusters, which is equal to the number of simultaneous speakers.

The elbow method is proposed for optimizing the classification analysis due to finding the optimal number of clusters based on the related distances. The main idea in the elbow algorithm is considering the elbow cost function as

(36) $$J=\sum_{i=1}^{K}\sum_{z\in C_{i}}{\left|z-C_{i}\right|}^{2}$$

where in Equation (36), *J* is the cost function, *z* is the data within cluster $C_{i}$, and *K* is the number of clusters $\left|C_{i}\right|$, where by increasing the number of clusters *K*, the sample partition is more modified. Furthermore, the degree of each cluster is increased, which means that the cost function *J* becomes smaller by increasing the *K* value. When *K* is less than the correct number of clusters, decreasing the *J* value is important, because increasing the *K* value greatly increases the degree of each cluster. In addition, when *K* reaches the real number of clusters, the clustering degree is highly decreased by increasing the *K* value. Therefore, the cost function *J* is sharply decreased and reaches the flat position, even by increasing the *K* value. Therefore, the relation between the cost function *J* and *K* values is defined as the elbow shape, and the related *K* value to the elbow point is considered as the correct number of clusters (speakers). Therefore, the proposed SB-2DASRP method in combination with unsupervised agglomerative clustering and elbow criteria is considered for estimating the number of simultaneous speakers based on the novel HNMA. The proposed method is compared with other previous works in undesirable scenarios, which is explained in the next section.

## 5. Results and Discussion

**Figure 7:**
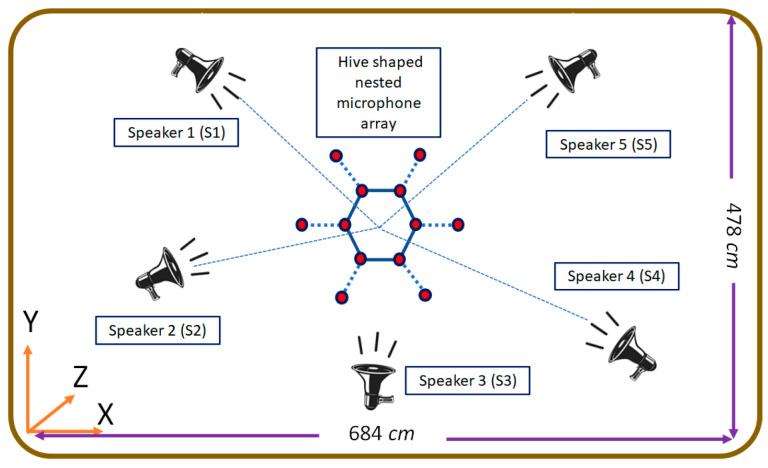
A view of the simulated room with the positions of HNMA and five simultaneous speakers.

**Figure 8:**
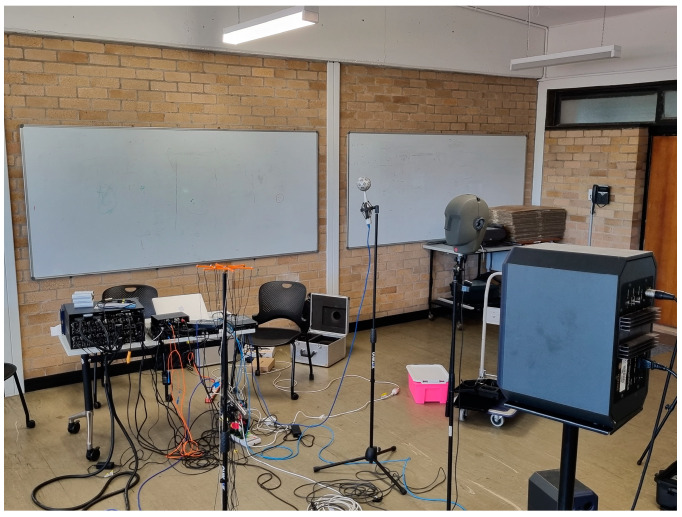
The acoustical room in the University of Sydney for real data recording.

For evaluating the performance, reliability, and precision of the speech processing algorithms, they were implemented on both real and simulated data. Therefore, the TIMIT dataset was considered for analysis of the proposed HNMA-SB-2DASRP method for preparing the simulated data [38]. The speech signals in the TIMIT dataset were recorded in various acoustical conditions for generating undesirable scenarios such as noise and reverberation. In addition, the proposed method was compared to other works based on real data, which were recorded in the computing and audio research laboratory at the university of Sydney, Australia. The simulated scenarios were considered as similar as possible to the real conditions, which makes the results comparable between these two environments. Since the proposed method estimated the number of simultaneous speakers, the simulations were implemented on the scenarios for two to five overlapped speakers, which normally happens in real recording conditions. More than five simultaneous speakers rarely appear in real scenarios. One male and one female speaker were selected for two simultaneous speakers’ conditions. Furthermore, a five overlapped speaker scenario included two female and three male simultaneous speakers. Figure 7 shows a view of the simulated room for recording the speech signal in speaker counting applications. The proposed algorithm was considered for a specific application of smart meeting rooms, where the speakers are around and at a short distance from a microphone array. As seen, the proposed HNMA was located at the center of the room for preparing the best spatial symmetry of microphone signals. Based on the defined scenarios for the evaluations, the room dimensions and the HNMA position were selected as (684, 478, 351) cm and (342, 239, 165) cm, respectively. In addition, the speakers’ locations were considered as S1 = (173, 382, 177) cm, S2 = (132, 203, 172) cm, S3 = (325, 64, 164) cm, S4 = (594, 96, 169) cm, and S5 = (523, 394, 179) cm, respectively, which was a proper distribution of the speakers in the acoustical room. Speakers could have been located in every place in the room, but this distribution of the speakers was proposed since it is a common scenario in normal meeting rooms. Based on the HNMA and speakers’ positions, the near-field criteria was a correct assumption for this scenario in simulations. In addition, Figure 8 shows the acoustical room in the University of Sydney for recording the real data.

**Figure 9:**
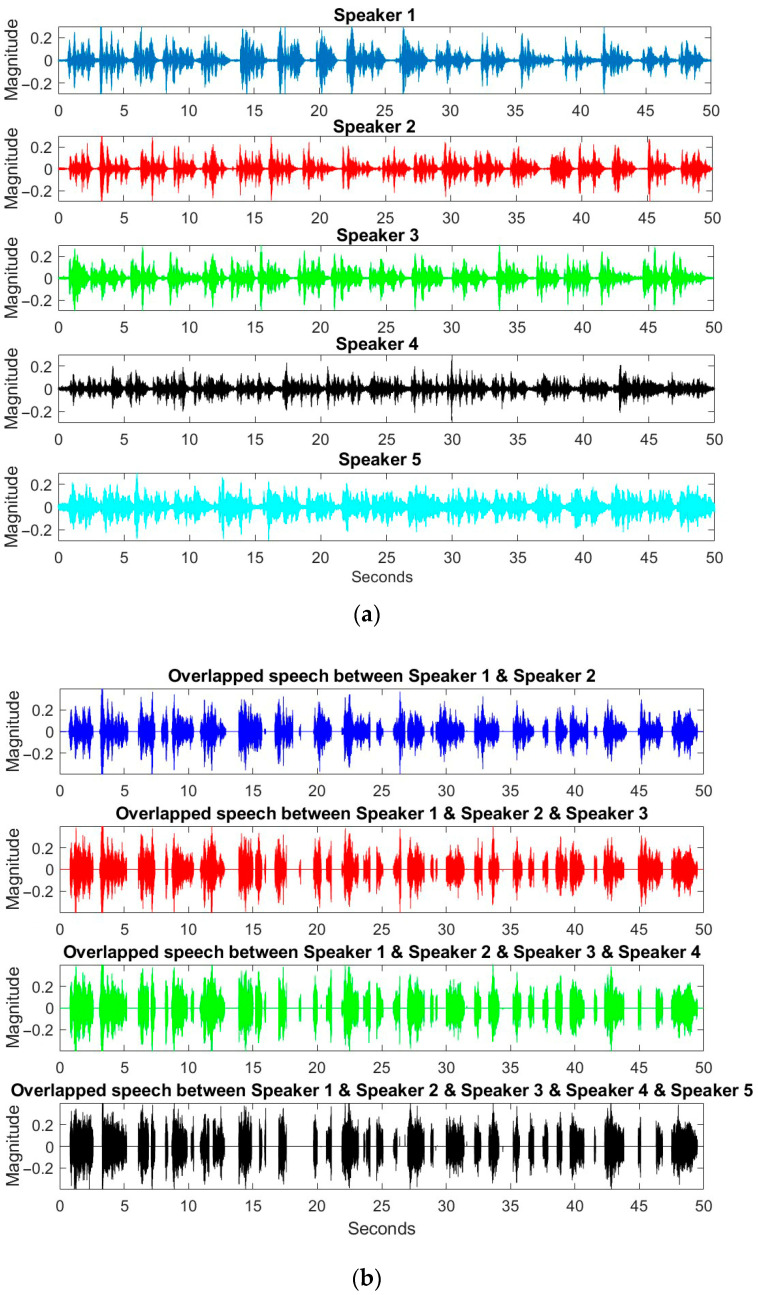
The speech signal for (**a**) each speaker and (**b**) the overlapped speech signals for two, three, four, and five simultaneous speakers in the simulations.

Since the techniques for speech signal processing were implemented in short time-frames, a Hamming window with 55 ms length and 50% overlap was selected for providing the maximum proper information for each speaker. Figure 9 shows the speech signal for each speaker and the overlapped signals for two to five simultaneous speakers, which were used in simulations of the proposed speaker counting algorithms. As seen, the length of the overlapped signals was decreased by increasing the number of simultaneous speakers, which clearly showed the lower probability of having many simultaneous speakers in the real scenarios. For the recorded data in the proposed scenario, 33.8 s of overlapped signal was related to the two simultaneous speakers, 29.2 s for three overlapped signals, 26.1 for four simultaneous speakers, and 24.2 s for five overlapped speakers, respectively.

The maximum frequency components and sampling frequency for the recorded signals were considered to be 8000 Hz and 16 kHz, respectively. Furthermore, noise, reverberation, and aliasing were the most undesirable factors, which decreased the quality of the recorded signals. The effect of aliasing was removed by the proposed HNMA, but the effect of the noise and reverberation were clearly observed in the recorded signals for the real scenarios. Therefore, simulated signals should be generated to be as similar as possible to the real recorded data. White Gaussian noise was selected as an additive noise in the microphones’ places, which accurately modeled the real environmental noise. Reverberation was another undesirable factor that decreased the accuracy of estimating the number of speaker methods. Various methods were proposed for simulating the effect of reverberation, where the image method was considered in the simulations of the proposed algorithm [39]. The image method produces the room impulse response between each speaker and microphone by considering the speaker position, room dimensions, sampling frequency, impulse response length, reverberation time ($RT_{60}$), and room reflection coefficients. The microphone signal is generated by convolution between the source signal and the produced room impulse response by the image method.

**Figure 10:**
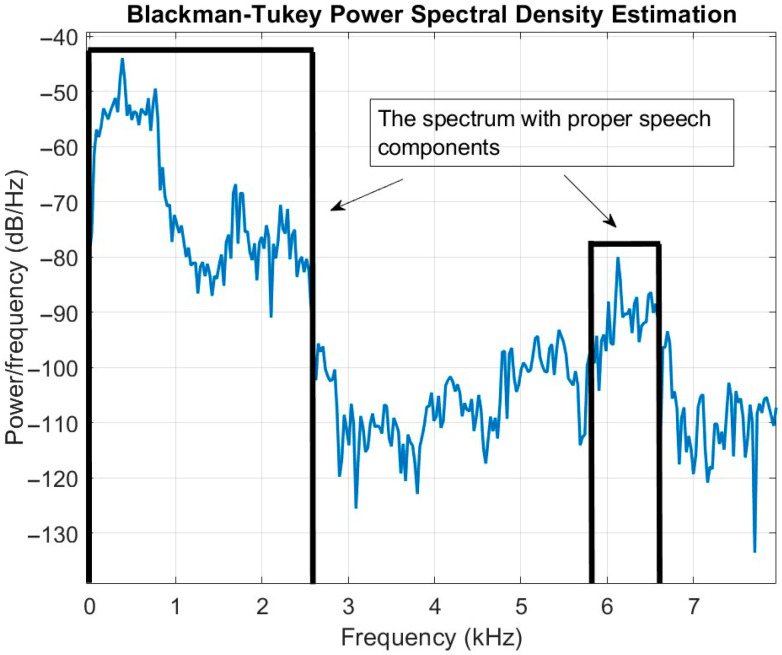
The spectral estimation of the speech signal by the Blackman–Tukey method based on the proposed threshold for eliminating the undesirable frequency components.

As was mentioned in Section 4.1, the Blackman–Tukey method was considered for evaluating the speech spectrum by selecting the proper frequency bands with highlighted speech signals, and by eliminating the frequencies with low components of the speech. This method was implemented on all recorded signals by HNMA to select the best part of the microphone signals. Figure 10 shows a sample spectral estimation by the Blackman–Tukey method and the selection of the proper spectral area on a frame of the recorded signal. As shown, in the frequency range [0–7800] Hz, the dominant speech components were in the frequency range [0–2657] Hz and [5871–6594] Hz, and in the rest of the signal, noise and reverberation were dominant. Therefore, a threshold as 30% of the maximum frequency amplitude of the signal was experimentally defined for selecting the proper frequency components and eliminating the undesirable frequencies. As shown, the desired frequency components of the speech signal were considered for the proposed speaker counting method, which increased the accuracy of the final results.

**Figure 11:**
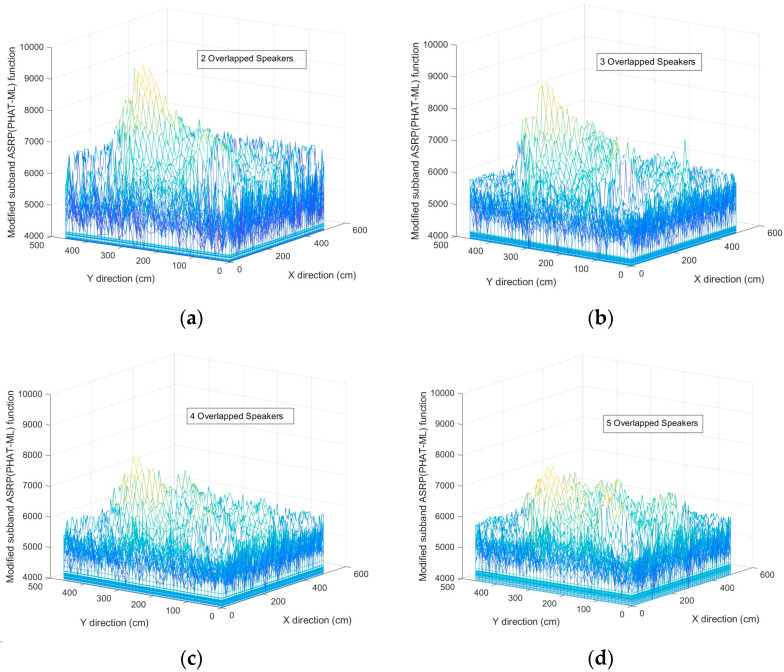
The energy map of the SB-2DASRP function for extracting the peak positions for (**a**) two, (**b**) three, (**c**) four, and (**d**) five simultaneous speakers.

In the following, the SRP method was adaptively implemented in 2D format on sub-band speech signals based on the PHAT and ML. The PHAT and ML filters were selected for the speech signals with SNR>10dB and SNR<10dB, respectively [35]. Therefore, the weighting filters prepared the best results for the sub-band-2DASRP algorithm. Figure 11 shows the energy map of the SB-2DASRP function in a fixed z value (z = 170 cm) due to sample conditions of two, three, four, and five simultaneous speakers in SNR=10dB and $RT_{60}=400\mathrm{ms}$. As seen, the SRP’s peaks related to the real speakers were affected by the environmental noise and reverberation. For example, in the two simultaneous speaker scenarios (Figure 11a), the peaks related to the speakers were seen more clearly, but for five simultaneous speakers (Figure 11d), the number of false peaks was exponentially increased because of the greater number of speakers. By increasing the number of speakers, the effect of false peaks was increased in the energy map of the SRP function and proportionally decreased the accuracy of estimations due to the increase in environmental reflections. Proposing the weighing filters, comparison with SD, classification, and elbow criteria were the steps used for decreasing the undesirable effects and for preparing more accurate data in order to estimate the final number of simultaneous speakers.

**Figure 12:**
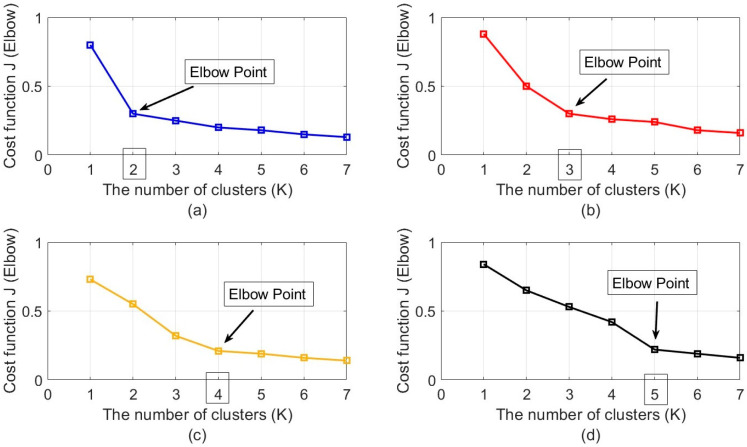
The elbow curves for estimating the number of speakers in various time frames of overlapped speech signals for (**a**) two, (**b**) three, (**c**) four, and (**d**) five simultaneous speakers.

The last step of the proposed system in Figure 5 is unsupervised classification in combination with elbow criteria for estimating the number of clusters (speakers). As was explained in Section 4.4, the number of clusters (speakers) was estimated based on the sharpness of changing the slope in the elbow curve. To select the correct number of speakers (*K*) based on the elbow curve, the slope of the curve was calculated for each *K* value. The slopes of the line before and after these points were calculated, and the point with the greatest difference between these slopes was automatically selected as the elbow. Figure 12 shows the elbow diagram for two to five simultaneous speakers in a sample scenario with a moderate level of noise and reverberation (SNR=10dB and $RT_{60}=400\mathrm{ms}$). As is seen, the slope of the elbow curve changed by increasing the *K* value, and suddenly the sharpness in the slope decreased in a specific *K* value. Figure 12a shows the sharp change in the slope for *K* = 2, which means that the number of simultaneous speakers was two in this dataset for classification in the specific time frames of the overlapped speech signal. Figure 12b–d similarly show the results for three, four, and five simultaneous speakers in various time frames, which clearly shows the benefits of elbow criteria for estimating the number of clusters (speakers). The large effects of noise and reverberation decreased the quality of the input data for the classification and indirectly the elbow criteria, but the results of the elbow method in the proposed system were more trustable due to the use of WPT and the implementation of the adaptive version of the SRP function based on PHAT and ML filters, which provided high-quality data for elbow criteria.

**Figure 13:**
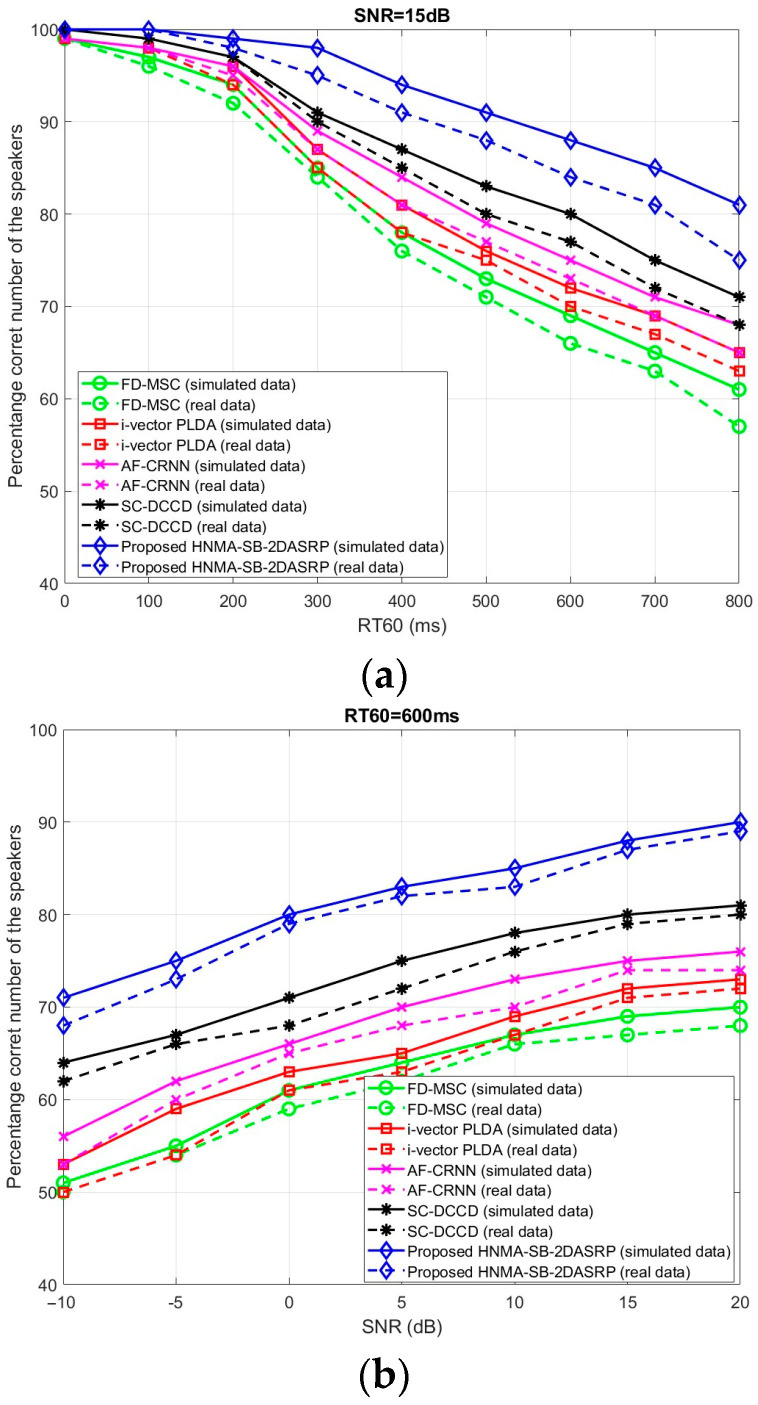
The percentage of correct number of speakers for 5 overlapped speech signals on real and simulated data for the proposed HNMA-SB-2DASRP method in comparison to the FD-MSC, i-vector PLDA, AF-CRNN, and SC-DCCD algorithms for (**a**) fixed SNR=15dB and variable $0\leq RT_{60}\leq 800\mathrm{ms}$, and (**b**) fixed $RT_{60}=600\mathrm{ms}$ and variable −10≤SNR≤20dB.

The proposed HNMA-SB-2DASRP method was compared to the FD-MSC [24], i-vector PLDA [25], AF-CRNN [26], and SC-DCCD [27] algorithms for two to five simultaneous speakers in the noisy and reverberant environments using real and simulated data. Two categories of the scenarios were considered for the evaluations. The first scenario was selected for evaluating the effects of the noise and reverberation separately for a fixed number of simultaneous speakers (five overlapped signals). Firstly, the noise was considered as a fixed value, and reverberation was variable, and secondly, the reverberation was fixed, and noise was variable. In the second category of the evaluations, all methods were evaluated for different numbers of simultaneous speakers on a high level of noise and reverberation as the worst acoustical conditions. Therefore, the results of the proposed system were reported based on the noise, reverberation, and the number of simultaneous speakers, separately. The percentage of the correct number of speakers was considered as the parameter for calculating the accuracy. Since the algorithm should be trustable and repeatable, only reporting the evaluations in one specific frame could not prepare the proper results. Therefore, we decided to repeat the proposed method on 100 frames and report the percentage of the correct number of speakers based on the obtained results. As was mentioned, the first category of the implementations was designed for evaluating the effect of the noise and reverberation. Figure 13a shows the results of the proposed HNMA-SB-2DASRP method in comparison with the FD-MSC, i-vector PLDA, AF-CRNN, and SC-DCCD algorithms for five simultaneous speakers in fixed SNR=15dB and $0\leq RT_{60}\leq 800\mathrm{ms}$ for real (dash line) and simulated (solid line) data. By considering the *SNR* as 15dB, the noise was practically eliminated, and the effect of the reverberation was evaluated on the proposed system. For example, in SNR=15dB and $RT_{60}=600\mathrm{ms}$ as a reverberant environment, the proposed HNMA-SB-2DASRP method estimated the correct number of speakers by 88% accuracy, the FD-MSC by 69%, the i-vector PLDA by 72%, the AF-CRNN by 76%, and the SC-DCCD by 80% using the simulated data for five simultaneous speakers. For the real data, the proposed HNMA-SB-2DASRP method estimated the correct number of speakers by 84%, FD-MSC by 66%, i-vector PLDA by 70%, AF-CRNN by 73%, and SC-DCCD by 78%, where the results were similar to the simulated scenario for five simultaneous speakers. As is seen, most of the methods lost accuracy in high reverberant scenarios, but the proposed HNMA-SB-2DASRP algorithm estimated the number of speakers with high accuracy. Figure 13b shows the comparison of the proposed HNMA-SB-2DASRP method to the FD-MSC, i-vector PLDA, AF-CRNN, and SC-DCCD algorithms for five simultaneous speakers in fixed $RT_{60}=600\mathrm{ms}$ and variable −10≤SNR≤20dB. Based on the mentioned scenario, the proposed HNMA-SB-2DASRP method was compared to other systems for five simultaneous speakers for reverberant and variable effects of the noise on real and simulated data. For example, in $RT_{60}=600\mathrm{ms}$ and SNR=5dB as a reverberant and noisy environment, the proposed HNMA-SB-2DASRP method estimated the number of correct speakers by 83% in comparison to 64% by the FD-MSC, 65% by i-vector PLDA, 70% by AF-CRNN, and 75% by SC-DCCD using the simulated data, which are shown with solid lines. In addition, this figure shows the results of the comparison of the real data between the proposed method and other research works. For example, in $RT_{60}=600\mathrm{ms}$ and SNR=5dB, the proposed HNMA-SB-2DASRP method estimated the number of speakers by 82%, which was more accurate in comparison to the 60% by FD-MSC, 63% by i-vector PLDA, 68% by AF-CRNN, and 71% by SC-DCCD, which shows the superiority of the proposed method in noisy scenarios in comparison to other systems. The results in this figure represent the better accuracy of the simulated results in comparison to the real data due to the impossibility of controlling the exact values of noise level and reverberation time ($RT_{60}$) in real recording scenarios. In addition, this figure shows that all methods estimated the number of simultaneous speakers with high accuracy in high *SNR* and low reverberation time ($RT_{60}$) conditions, but the accuracy was decreased by adding more noise and reverberation effects. In contrast, the proposed method estimated the number of simultaneous speakers with more reliability in comparison to previous works.

**Figure 14:**
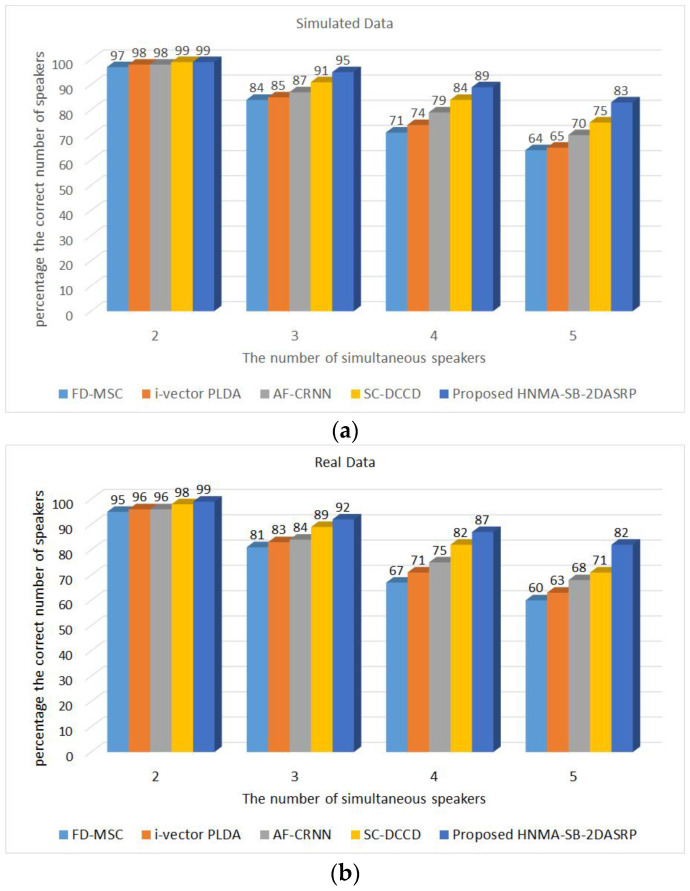
The percentage of the correct number of speakers for the proposed HNMA-SB-2DASRP method in compassion with the FD-MSC, i-vector PLDA, AF-CRNN, and SC-DCCD algorithms in noisy and reverberant environments (SNR=5dB and $RT_{60}=600\mathrm{ms}$) for 2 to 5 simultaneous speakers for (**a**) simulated data and (**b**) real data.

The second category of the experiments was conducted for evaluating the proposed method on a variable number of speakers in noisy and reverberant scenarios. Figure 14a shows a comparison of the proposed HNMA-SB-2DASRP method to the FD-MSC, i-vector PLDA, AF-CRNN, and SC-DCCD algorithms in noisy and reverberant scenarios (SNR=5dB and $RT_{60}=600\mathrm{ms}$) for two to five simultaneous speakers using simulated data. This comparison was structured for evaluating the accuracy of each algorithm for various number of simultaneous speakers. For example, in the two simultaneous speakers scenario, the proposed HNMA-SB-2DASRP method estimated the correct number of speakers by 99% in comparison to the FD-MSC by 97%, i-vector PLDA by 98%, AF-CRNN by 98%, and SC-DCCD by 99%. These results show that most of the methods estimated the correct number of speakers with high accuracy in a small number of overlapped speech signals, even in noisy and reverberant conditions, due to the low interference and reverberation of the speech signal. For four simultaneous speakers, the proposed HNMA-SB-2DASRP method estimated the correct number of speakers by 89% in comparison to the FD-MSC by 71%, i-vector PLDA by 74%, AF-CRNN by 79%, and SC-DCCD by 84%, which shows the superiority of the proposed method with high numbers of simultaneous speakers. Figure 14b similarly shows the results of the comparison between the proposed HNMA-SB-2DASRP method to the FD-MSC, i-vector PLDA, AF-CRNN, and SC-DCCD algorithms on the real data. For example, with two overlapped speakers, the percentage of the correct number of speakers for the proposed method was 99% in comparison to the FD-MSC by 95%, i-vector PLDA by 96%, AF-CRNN by 96%, and SC-DCCD by 98%, which shows the close results of all methods for a small number of simultaneous speakers using real data, which was similar to the results using simulated data. In addition, the methods were compared for a higher number of simultaneous speakers to show the effects of adding more speakers in the environment. For example, in four simultaneous speakers, the proposed method estimated the number of speakers by 87% accuracy in comparison to the FD-MSC by 67%, i-vector PLDA by 71%, AF-CRNN by 75%, and SC-DCCD by 82%. As seen, the accuracy of all previous methods was decreased by increasing the number of simultaneous speakers, but the proposed HNMA-SB-2DASRP algorithm estimated the number of overlapped speakers with more accuracy and reliability in comparison to other systems in noisy and reverberant scenarios with a high number of speakers, which shows the superiority of the proposed speaker counting system in comparison to the previous works.

Finally, Table 1 represents the computational complexity of the proposed HNMA-SB-2DASRP method in comparison to the FD-MSC, i-vector PLDA, AF-CRNN, and SC-DCCD algorithms. The computer programming run-time in seconds was selected as a factor for measuring and comparing the complexity between the algorithms. The experiments were implemented by MATLAB software on a laptop with a i7-10875H CPU core (Intel, Santa Clara, CA, USA), 2.3 GHz, and 64 GB RAM. In addition, the results were extracted for noisy (SNR=5dB and $RT_{60}=200\mathrm{ms}$), reverberant (SNR=15dB and $RT_{60}=600\mathrm{ms}$), and noisy–reverberant (SNR=5dB and $RT_{60}=600\mathrm{ms}$) scenarios for five simultaneous speakers using real and simulated data. As seen, the AF-CRNN algorithm contained the highest computational complexity in comparison to other works due to the use of neural networks for training and testing steps. For example, on simulated data, the programming run-time for the proposed HNMA-SB-2DASRP method was 32 s in comparison to the FD-MSC by 52 s, i-vector PLDA by 44 s, AF-CRNN by 78 s, and SC-DCCD by 42 s in noisy and reverberant environments, which showed the low computational complexity of the proposed method in comparison to the other works. The results using the real data similarly showed the superiority of the proposed HNMA-SB-2DASRP method in comparison to previous research. For example, with real data, the proposed method had a programming run-time of 36 s in comparison to that of the FD-MSC of 54 s, i-vector PLDA of 47 s, AF-CRNN of 85 s, and SC-DCCD of 39 s in noisy and reverberant environments. Therefore, the proposed HNMA-SB-2DASRP method is considered as a proper instrument for estimating the number of simultaneous speakers in noisy and reverberant environments due to the high accuracy and reliability in the experiments and low computational complexity of the implementations.

## 6. Conclusions

Speaker counting is an important area in speech signal processing related to smart meeting rooms, especially as a preprocessing step for other applications such as speech enhancement, speaker separation, sound source localization, and speaker tracking. Undesirable environmental factors such as noise, reverberation, and aliasing highly decrease the accuracy of the estimations in most of the common algorithms. In this article, a novel speaker counting system is presented based on the proposed HNMA in combination with WPT for smart signal processing. In the following, a 2D and sub-band version of the SRP function adaptively prepares the required data for unsupervised agglomerative classification with elbow criteria for estimating the number of simultaneous speakers. The novel HNMA is proposed not only to prepare the spatial symmetry of data recording but also to be implemented in other applications based on its capacity of spatial extension. Then, the WPT method is proposed for sub-band processing and preparing high frequency resolution on low frequency components and proper frequency resolution on high frequency components. In the next step, an adaptive SRP function in 2D format by use of PHAT and ML filters is adaptively implemented on each sub-band by WPT. The extracted peak positions in this function are weighted based on the proposed weighing coefficients due to the largest peak in each sub-band. Finally, SD criteria are selected for eliminating improper information and preparing suitable data for clustering. In the last step, the unsupervised agglomerative algorithm in combination with elbow criteria is considered for classifying the obtained data of the last step and estimating the optimal number of clusters (speakers). The proposed HNMA-SB-2DASRP method is compared to the FD-MSC, i-vector PLDA, AF-CRNN, and SC-DCCD algorithms on noisy and reverberant scenarios for real and simulated data, where the results show the superiority and high accuracy of the presented method in comparison to previous works for estimating the number of simultaneous speakers in smart meeting room applications. In addition, computational complexity was calculated based on the programming run-time, where the proposed HNMA-SB-2DASRP algorithm estimates the NoS with less complexity in comparison to the other methods. Therefore, the obtained results on real and simulated data represent the superiority of the proposed HNMA-SB-2DASRP algorithm for estimating the NoS in comparison to the previous traditional research works.

## Figures and Tables

**Table 1:** A comparison of the computational complexity of the proposed HNMA-SB-2DASRP method and the FD-MSC, i-vector PLDA, AF-CRNN, and SC-DCCD algorithms on real and simulated data based on MATLAB programming run-time (seconds) in noisy and reverberant environments for 5 simultaneous speakers.

Computational Complexity (Program Run-Time (s))					
	Simulated Data				
**Scenario**	FD-MSC	i-vector PLDA	AF-CRNN	SC-DCCD	Proposed HNMA-SB-2DASRP
Noisy	43	35	64	37	21
Reverberant	46	36	69	34	26
Noisy–Reverberant	52	44	78	42	32
	**Real Data**				
**Scenario**	FD-MSC	i-vector PLDA	AF-CRNN	SC-DCCD	Proposed HNMA-SB-2DASRP
Noisy	48	42	69	38	25
Reverberant	51	48	73	36	29
Noisy–Reverberant	54	47	85	39	36

## Data Availability

Not applicable.

## Abbreviations

The following abbreviations are used in this manuscript:

2DASRP	Two-dimensional adaptive steered response power
AF-CRNN	Ambisonics features of the convolutional recurrent neural networks
CWT	Continues wavelet transform
DWT	Discrete wavelet transform
FD-MSC	Frequency domain magnitude square coherence
FSB	Filter-and-sum beamformer
HNMA	Hive shaped nested microphone array
HPF	High-pass filter
LPF	Low-pass filter
ML	Maximum likelihood
NoS	Number of speakers
PHAT	Phase transform
PLDA	Probabilistic linear discriminant analysis
SC-DCCD	Speaker counting based on density clustering and classification decision
SD	Standard deviation
SRP	Steered response power
SSL	Sound source localization
TF	Time–frequency
WPT	Wavelet packet transform
